# Moral Injury, Emotional Labor and Burnout: An Unbearable Burden for Health Professionals

**DOI:** 10.12669/pjms.37.5.4794

**Published:** 2021

**Authors:** Chaudhry Aqeel Safdar, Rashid Qayyum

**Affiliations:** 1 Chaudhry Aqeel Safdar, FRCSEd, MSc, MCPS-HPE Fazaia Medical College & PAF Hospital, Air University, Islamabad - Pakistan; 2 Rashid Qayyum, FCPS (Psych), MHPE Fazaia Medical College & PAF Hospital, Air University, Islamabad - Pakistan

Medical students and young trainees embark on the journey to become doctors with idealism and enthusiasm for curing disease and infirmity and improving their patients’ life. Despite the intention of most medical schools to nurture these qualities, it is ironic that many studies, from Pakistan and abroad report a decline in qualities like humanitarianism, enthusiasm and idealism.^1^ We all signed up for this job to be healers, to help people, but something gets lost along the way. In the early years of medical school, we are taught about treating the patient as a “whole”, with a humanistic and empathetic approach. But in final years of medical school and later in clinical training we’re encouraged to quickly adopt a new reality: the dehumanization of medicine. We check boxes, treat lab values, and objectify patients instead of learning how to effectively heal them.^2^ The competitive nature of medical studies and lack of such training at medical schools are also contributing factors behind this decline.^3^

There is a need to understand what goes wrong which leads to this change in the behavior and attitudes of the ‘healers’. Could it be a sign of moral injury and emotional labor? Physicians on the front lines of health care these days are sometimes described as going to battle. It’s an apt metaphor. Health professionals, like soldiers, may have to also contend with significant threats to their well-being: ***moral injury***.^4^ It represents “perpetrating, failing to prevent, bearing witness to, or learning about acts that transgress deeply held moral beliefs and expectations” first described by Silver.^5^

Moral injury is frequently mischaracterized. Among physicians it is portrayed as burnout. But without understanding the critical difference between burnout and moral injury, the wounds will never heal and physicians and patients alike will continue to suffer the consequences. ***Burnout*** is a constellation of symptoms that include malaise, exhaustion, frustration, cynicism, and decreased productivity; this arises from “making excessive demands on energy, strength, or resources” in the workplace.^6^ More than half of physicians report at least one of these. Health care workers during COVID pandemic performed their duties putting them under tremendous risk by working with inadequate personal protective equipment and working long hours without break, fighting a battle to see little improvement, sometimes because of limited resources and lack of clarity how to save them from the pain of death. This is a huge emotional burden.

Health professionals internalize the burnout poorly. It somehow, for them, equates to some sort of failure in their job. It seems to them that they have failed their training and they are “weak”. ***We believe that burnout is itself a symptom of something larger: our broken health care system*.** The increasingly complex web of providers’ highly conflicted allegiances — to patients, to self, to supervisors and to employers — and its attendant moral injury may be driving the health care ecosystem to a tipping point and causing the collapse of resilience.^4^

Most doctors enter medicine following what seems like a higher calling, and not an ordinary career. There is a desire to help people. Many enter it with almost religious zeal, enduring lost sleep, lost youth years, family strain, financial loss, ignoring personal health, and many more challenges. Each hurdle increases their perseverance towards the sharply focused goal of best care for one’s patients, alleviating their suffering. Failing to consistently meet patients’ needs has a profound impact on their wellbeing — this is the basis of the ensuing moral injury.

***Emotional labor*** refers to the “enhancing, faking, or suppressing emotions” to fulfill the requirements of a job or situation. This may be when we are looking calm while anxiously awaiting some sort of abuse from the patients and seniors, keeping a smile on the face, or the cumulative emotional toll of always being “nice” throughout duties.

Emotional labor and moral injury brings on a feeling when on so many occasions throughout the duties we wished a patient received better treatment and when we feel powerless. This is so often seen in COVID pandemic, as witnessed in the third wave in India. It isn’t easy when a patient is brought by his family begging for oxygen support and you have to deny admission because of non-availability of bed. More painful would be when you have to triage putting patients on ventilators knowing fully well that some of them would die just because of non-availability of the ventilators. Or you might have to discharge patients earlier to create beds for new patients.^7^ This makes you acutely vulnerable to moral injury. This is not only seen in doctors but nurses also, who have witnessed these difficult ethical situations in Covid patients and when faced with unnecessary patient suffering and a feeling of not doing enough.^8^

In day to day routine, it is the anger that you may feel when told to manipulate to get a “difficult” patient to leave the hospital or shifted. It’s the shame you feel when a senior labels our patient as malingering, when may be a little more patience, communication, and open-mindedness would have found a better answer. It’s the sadness, anger, and resentment that these young idealistic doctors feel every time you hear, “I like it better when the patient is not in bed because then I don’t have to talk to him.”

These experiences all have an emotional toll. Every time these doctors try to treat a patient with empathy and compassion, they may be made to feel bad or embarrassed about it *(“You’re being too nice, the patient and relatives will make your life hell” “This patient is a waste of time.” “Forget it; patients like this can’t be helped.”)*. Every time a tiny piece of their soul fractures, and fades off. They wonder if these seniors were like them once, full of optimism and high ideals to help people. Is this what happens to a person after repetitive moral injury? “Should I continue to strive to be a good person and a good doctor?”

This is where the emotional labor comes in. Being constantly subjected to the inhumanities and indignities of medicine while keeping a good attitude and smile on one’s face is exhausting. Add on the expectation to be constantly studying for specialization and guilt when they are too tired to do so, and it’s a recipe for depression and anxiety, and unfortunately suicidal inclinations. It is not surprising why mental health among medical students and physicians is so poor.^2^

In an increasingly business-oriented and profit-driven health care environment, doctors must consider a multitude of factors other than their patients’ best interests when deciding on treatment. Financial considerations — of hospitals, health care systems, insurers, patients, and sometimes of the physicians themselves — lead to conflicts of interest. They can also prevent doctors from providing necessary but unwelcome advice to patients, and can lead to over-treatment to keep some patients satisfied.^4^ This moral injury is an insult to a person’s moral conscience or values. It damages or transgresses his or her “deeply held moral beliefs and expectations.” When these transgressions happen, they can produce a wide range of emotions like guilt, shame, sadness, betrayal, or anger, a recipe for moral injury.^2^

Continually being caught between the oath they took, long years of training, and the realization of making a profit from sick people when they are most vulnerable is an untenable and unreasonable demand. Routinely experiencing the suffering, anguish, and loss of being unable to deliver the care that patients need is deeply painful, for these young idealists. These routine, betrayals of patient care and trust are examples of “death by a thousand cuts”.^4^

In order to ensure that these compassionate young health professionals are providing best patient care, executives must recognize and acknowledge that this is not physician burnout. Simply holding lectures and workshops won’t solve the problem. Nor will getting physicians to practice mindfulness, meditation, and relaxation techniques. None of these measures is geared to change the hospital practices that inflict moral injuries.

What we need is health leadership willing to understand the human costs and moral injury of multiple competing interests. Physicians must be treated with respect, autonomy, and the authority to make evidence-based, ethical and financially responsible decisions. Hierarchal authoritarian mandates on medical practice are debasing and ultimately ineffective.

We also need administrators and clinical leaders who recognize that caring for their health professionals’ results in thoughtful, compassionate care for patients, which ultimately is good business. Senior doctors’ experience and skills are beyond petty business gains and could be treated with loyalty and not as a replaceable asset. A little appreciation, along with constructive feedback and encouragement goes a long way. The patients also need to be sensitized to the power of appreciation and an occasional thank you. All this should be without the best interest of the business of health care. These goals should be aimed at creating a win-win. This is the best way to avoid the ongoing moral injury and emotional labor associated with health care.
